# Combined Endodontic Retreatment and Intentional Replantation for External Cervical Resorption: A Four-Year Follow-Up Case Report

**DOI:** 10.3390/reports9040328

**Published:** 2026-09-28

**Authors:** Ayman M. Abulhamael

**Affiliations:** Department of Endodontics, Faculty of Dentistry, King Abdulaziz University, P.O. Box 80209, Jeddah 21589, Saudi Arabia; amahmad4@kau.edu.sa; Tel.: +966-598977241

**Keywords:** external cervical resorption, ECR, intentional replantation, root canal retreatment

## Abstract

**Background and Clinical Significance:** External cervical resorption (ECR) is an insidious form of root resorption that frequently progresses undetected until an advanced stage, particularly when the lesion extends apical to the alveolar crest or involves a substantial proportion of the root circumference, precluding adequate visualization and debridement via conventional intraoral approaches. **Case Presentation:** This report describes the management of a 30-year-old male who presented with an acute apical abscess associated with a mandibular second molar with inadequate previous root canal treatment, in conjunction with an extensive distal cervical resorption defect and a large periapical radiolucency. Due to lack of availability of a CBCT machine at the time, no preoperative CBCT was obtained; diagnosis and treatment planning were based on clinical examination and periapical radiography. Given the anatomical inaccessibility of the resorptive lesion and the concurrent persistent periapical pathology, treatment consisted of nonsurgical root canal retreatment followed by intentional replantation to permit extraoral access and repair. The tooth was replanted after an extraoral period of less than 15 min and stabilized with a flexible splint. The patient was reviewed at 2 weeks and was then lost to routine follow-up until a 4-year reassessment. At that time, the tooth was functional and asymptomatic, with complete radiographic resolution of the previous periapical radiolucency and no clinically or radiographically evident signs of progressive root resorption or ankylosis. **Conclusions:** This case documents a favorable four-year outcome following intentional replantation combined with endodontic retreatment and bioceramic repair in a multi-rooted tooth with extensive, conventionally inaccessible ECR.

## 1. Introduction and Clinical Significance

External cervical resorption (ECR) is an uncommon and potentially destructive form of external root resorption that originates on the external surface of a permanent tooth in the cervical region [1]. The condition is characterized by progressive loss of cementum and dentin and may extend coronally beneath the enamel, apically along the root, circumferentially around the tooth, and internally toward the root canal system [1]. Because of this pattern of spread, ECR is often detected incidentally during routine clinical or radiographic examination, or only after the lesion has already reached an advanced stage [1]. The etiology of ECR remains incompletely understood; damage to, or absence of, the protective cemental layer is believed to expose the underlying mineralized tissues to clastic cells and to facilitate initiation of the resorptive process [1]. Orthodontic treatment, traumatic dental injuries, intracoronal bleaching, periodontal procedures, and dentoalveolar surgery have been reported as predisposing factors, although ECR can also occur without an identifiable factor, supporting the view that its pathogenesis is multifactorial and still only partially understood [1,2,3]. Accurate three-dimensional assessment of the lesion is crucial for diagnosis, treatment planning, and management of ECR [4]. Patel classification records lesion height (1–4), circumferential spread (A–D), and proximity to the root canal (d, confined to dentine; p, probable pulpal involvement) (Figure 1) [4]. Because periapical radiography may underestimate lesion size and circumferential spread, CBCT can materially alter assessment and treatment planning in ECR [5]. The 2023 European Society of Endodontology position statement similarly recommends justified use of CBCT when three-dimensional information is expected to aid diagnosis or management [3].

Clinical management of ECR aims to eliminate or inactivate the resorptive tissue, seal the portal of entry, restore the resulting defect, and preserve pulpal and/or periodontal health whenever possible [1,3]. Treatment selection should consider not only lesion extent but also accessibility, probing feasibility, symptoms, remaining tooth structure, periodontal implications, and the relationship of the lesion to the root canal [6,7]. Management may include external nonsurgical debridement, periodontal surgery, internal repair with root canal treatment, orthodontic extrusion, intentional replantation, extraction, or a combination of these procedures, depending on the location, accessibility, and extent of the lesion [6,7]. However, lesions on inaccessible proximal or palatal surfaces, extending deeply below the alveolar crest, or involving a substantial portion of the root circumference can be difficult to visualize, debride, and restore through conventional intraoral approaches [1]. Incomplete elimination of the resorptive tissue may allow the disease to progress and further tooth structure to be lost, whereas overly aggressive removal of sound tissue may compromise the tooth’s restorability and long-term prognosis [1]. Intentional replantation involves the controlled extraction of a tooth, extraoral evaluation and treatment of the root surface or root canal system, and subsequent replacement of the tooth into its original socket [8,9]. The procedure has traditionally been regarded as a treatment of last resort. It can provide direct access when an external surgical or internal approach is impractical, but the evidence base specifically for ECR remains limited and is dominated by case reports and case series; therefore, it should be described as a selected tooth-preserving option rather than a universally predictable treatment [7].

The long-term outcome of intentional replantation depends primarily on preserving the vitality of periodontal ligament cells during extraction, extraoral manipulation, and reinsertion of the tooth [8,10,11]. Intentional replantation protocols emphasize atraumatic crown-level handling, avoidance of unnecessary root surface manipulation, maintenance of root surface moisture, efficient extraoral treatment, and appropriate stabilization when clinically required [8,10,11]. Using intentional replantation for ECR provides direct visual and operative access to root surfaces that may otherwise be inaccessible through conventional periodontal or restorative surgery [8,10]. Extraoral access may permit more accurate identification of the portal of entry and the circumferential extent of the lesion, complete removal of the resorptive tissue, and restoration of the defect under magnification [8]. However, successful application of this technique requires sufficient remaining tooth structure, acceptable periodontal support, a predictable extraction pathway, and the ability to complete extraoral repair within a limited period while preserving periodontal ligament viability [8,11]. Modern calcium-silicate-based sealers and repair materials are widely used in endodontics because of favorable handling and biological characteristics; however, the term ‘bioceramic’ encompasses different formulations, and biological or physical properties should not be generalized across products without material-specific evidence [12]. This case report describes the management of an extensive, conventionally inaccessible external cervical resorption lesion using a combination of nonsurgical root canal retreatment and intentional replantation, extraoral debridement and restoration of the resorptive defect, and subsequent clinical and radiographic follow-up.

## 2. Case Presentation

A 30-year-old male patient presented to the clinic with pain and swelling in the left posterior mandibular region. His medical history was noncontributory, and he was otherwise fit and healthy. The patient reported that root canal treatment had been performed on the mandibular left second molar (tooth #37) approximately 4 years earlier. Pain associated with the tooth had begun 5 days before presentation and had increased in intensity over the preceding 24 h, coinciding with the development of localized non-fluctuant swelling. Extraoral examination showed normal facial symmetry, no lymphadenopathy, and no fever reported. Clinical examination revealed a fractured coronal restoration and localized buccal swelling adjacent to tooth #37. The tooth was tender to percussion and palpation. Pulp sensibility testing was not applicable, as the tooth had previously undergone root canal treatment. Tooth mobility was within physiologic limits of a grade 0 Miller classification, and periodontal probing depths were within normal limits, measuring 3, 2, and 3 mm at the mesiobuccal, mid-buccal, and distobuccal sites, respectively, and 3, 2, and 3 mm at the mesiolingual, mid-lingual, and distolingual sites, respectively. An intraoral periapical radiograph revealed an inadequately performed previous root canal treatment and loss of integrity of the coronal restoration. An irregular radiolucent defect was observed on the distal cervical aspect of the tooth, extending apically below the level of the alveolar crest and suggesting external cervical resorption. A large periapical radiolucency was also associated with the roots of tooth #37 (Figure 2). Due to the unavailability of a CBCT machine at the time and location, the lesion could not be completely characterized preoperatively according to the three-dimensional Patel classification because circumferential spread and precise proximity to the canal could not be reliably determined from the periapical image alone [3,4,5]. The available findings nevertheless demonstrated a subcrestal distal ECR defect with internal communication encountered during treatment. Based on the clinical and radiographic findings, a diagnosis of a previously treated tooth with an acute apical abscess was made. All available treatment options, along with their potential advantages, limitations, and prognoses, were explained to the patient. These options included no active treatment; nonsurgical root canal retreatment with repair of the resorptive defect, if accessible; and nonsurgical root canal retreatment followed by intentional replantation to permit extraoral repair of the inaccessible resorptive defect, with extraction of the tooth as an alternative if retention was not feasible. Intentional replantation was explained to be the best option because the distal subcrestal location and limited intraoral access were considered unlikely to permit complete visualization, debridement, and restoration of the defect without potentially extensive periodontal surgical access. After discussing the available options, the patient elected to undergo nonsurgical root canal retreatment followed by intentional replantation. He was informed that extraction would be performed if the tooth could not be predictably treated or retained. Written informed consent was obtained before treatment.

### 2.1. First Treatment Visit

Local anesthesia was administered using an inferior alveolar nerve block with 2% lidocaine containing 1:100,000 epinephrine. The tooth was isolated with a rubber dam, the existing coronal restoration was removed, and endodontic access was established. The resorptive defect was exposed internally. The portion of the resorptive tissue that was directly accessible from the internal aspect was removed to reduce the accessible tissue burden and permit hemostasis, while recognizing that the subcrestal distal component could not be completely visualized or debrided in situ. The existing gutta-percha filling material was removed from the mesiobuccal, mesiolingual, and distal canals. The working length was determined using a Root ZX electronic apex locator (J. Morita Mfg. Corp., Kyoto, Japan). However, apical patency was achieved only in the distal canal; patency could not be established in either mesial canal. Pus drainage was noted from the distal canal. The canals were mechanically cleaned and irrigated with 20 mL of 5.25% sodium hypochlorite and 10 mL of 17% EDTA. Manual activation was used in the irrigation of sodium hypochlorite followed by 1 min irrigation of 17% EDTA. Calcium hydroxide paste (Metapaste; Meta Biomed Co., Ltd., Cheongju, Republic of Korea) was placed in the canals as an intracanal medicament for 2 weeks, and the access cavity was provisionally restored with a cotton pellet and Fuji IX glass-ionomer cement. A postoperative radiograph was obtained to verify placement of the calcium hydroxide dressing (Figure 3). The patient was discharged in stable condition and instructed to contact the clinic if pain, swelling, or any other complications developed before the next scheduled appointment.

### 2.2. Second Treatment Visit

At the second visit the patient was asymptomatic, with no swelling or tenderness to percussion or palpation. Local anesthesia was administered using an inferior alveolar nerve block with 2% lidocaine containing 1:100,000 epinephrine, and the tooth was isolated with a rubber dam. The provisional restoration and cotton pellet were removed, and the calcium hydroxide dressing was flushed from the canals using 15 mL of 5.25% sodium hypochlorite and 10 mL of 17% EDTA. The internal aspect of the resorptive defect was temporarily blocked with polytetrafluoroethylene (Teflon) tape to prevent extrusion of materials during canal preparation and obturation. The mesiobuccal and mesiolingual canals were prepared to size F2 using ProTaper Gold instruments (Dentsply Sirona, Charlotte, NC, USA); however, apical patency could not be achieved in either canal. The precise cause of the obstruction could not be definitively established at treatment; an obstruction associated with the previous root canal treatment and/or a pre-existing procedural alteration was considered possible. The distal canal was prepared to size F3 using the same instrumentation system. The canals were dried with sterile paper points and obturated with gutta-percha and CeraSeal bioceramic sealer (Meta Biomed, Cheongju, Republic of Korea). The internal aspect of the resorptive defect was repaired with bioceramic putty (CeraPutty; Meta Biomed, Cheongju, Republic of Korea). The coronal access was restored with composite resin. Because the apical termini of the mesial canals could not be predictably negotiated and a large periapical lesion was present, root-end management was planned during the extraoral phase. Intentional replantation was then performed during the same appointment.

### 2.3. Intentional Replantation Procedure

The tooth was atraumatically extracted using dental forceps. No dental elevator was used; the forceps was placed on the crown to avoid any root damage or damage to the PDL. After luxation of the tooth and before removal from the socket and to reduce the risk of accidental slippage during extraoral manipulation, two heavy rubber bands were secured around the forceps. After successful removal of the tooth without any crown or root fracture, direct manipulation of the root surface was minimized to preserve the periodontal ligament. Throughout the extraoral procedure, the root surfaces were continuously irrigated with Pedialyte solution to maintain hydration and minimize desiccation of the periodontal ligament; it is not presented as an established or superior periodontal ligament storage medium. Contemporary intentional replantation literature instead emphasizes minimizing extraoral dry time and maintaining the root surface in a physiologically compatible moist environment [11,12].

After extraction, the distal cervical resorptive defect was directly visualized. Residual resorptive tissue was mechanically removed using a size 4 round bur in a high-speed handpiece under microscopic magnification, with Pedialyte used for irrigation. After complete removal of the resorptive tissue, the cavity was examined under high magnification to confirm complete removal of the resorptive tissue; then, the defect was restored externally with bioceramic sealer and putty (CeraPutty; Meta Biomed, Cheongju, Republic of Korea) (Figure 4a). Subsequently, a 3-mm root-end resection of both the mesial and distal roots was performed using #557 surgical-length fissure carbide burs at a zero-degree bevel, followed by retrograde preparation to a depth of 3 mm using a #330 bur (Figure 4b). Then, retrograde cavities were filled with bioceramic putty (CeraPutty; Meta Biomed, Cheongju, Republic of Korea) (Figure 4c).

The tooth was maintained extraorally for less than 15 min before being gently repositioned into its original socket using digital pressure. The patient was asked to close his jaw to facilitate complete seating of the tooth within the alveolus, and then to bite on a cotton roll for 5 min. The replanted tooth was stabilized with a flexible splint using stainless steel 0.014 wire extending from tooth #36 to tooth #38, and a radiograph was taken (Figure 4d). The patient was advised to follow a soft diet, avoid chewing on the treated side for 5 days, and maintain appropriate oral hygiene. Ibuprofen 400 mg was prescribed for pain control, together with a 0.2% chlorhexidine gluconate mouth rinse. Amoxicillin-clavulanic acid, 1 g twice daily for 7 days, was prescribed as a case-specific perioperative decision in the context of the tooth’s initial acute apical abscess and the replantation procedure. At the second visit, the patient had no swelling or other reported systemic signs; therefore, this prescription should not be interpreted as evidence that systemic antibiotics are routinely required after intentional replantation.

### 2.4. Follow-Up and Outcome

The patient returned 2 weeks after intentional replantation for splint removal. Tooth #37 was clinically stable, with no abnormal mobility, pain, swelling, or tenderness to percussion or palpation (Figure 5a). The flexible splint was removed, and the patient was advised of the importance of a definitive cuspal coverage restoration.

The patient was subsequently lost to routine follow-up and did not undergo continuous longitudinal review; he returned for a late reassessment 4 years after treatment. At that visit, tooth #37 remained in the oral cavity, was fully functional, and was asymptomatic. Clinical examination revealed no swelling, tenderness, or abnormal mobility (grade 0 Miller classification), periodontal probing depths were within normal limits, measuring 3 mm at all six sites: mesiobuccal, mid-buccal, distobuccal, mesiolingual, mid-lingual, and distolingual, gingival condition was excellent, and there was no high metallic percussion sound or presence of sinus tract. Although the patient had not obtained the recommended full-coverage crown, the tooth remained functional. A follow-up periapical radiograph showed disappearance of the previous periapical radiolucency with re-establishment of a satisfactory periradicular trabecular pattern and periodontal ligament/lamina dura appearance. These findings were used as the qualitative criteria for complete radiographic healing (Figure 5b). Ankylosis was assessed using the available clinical findings (mobility and percussion) together with the periapical radiograph; accordingly, the outcome is reported as showing no clinically or radiographically evident signs of ankylosis or progressive root resorption. Limited areas of ankylosis or replacement resorption cannot be definitively excluded by a two-dimensional periapical radiograph. The clinical course and key treatment events are summarized in Table 1.

## 3. Discussion

This case demonstrates the successful management of a previously treated mandibular second molar presenting with an acute apical abscess, a large periapical lesion, and an extensive distal external cervical resorption defect, achieved by combining nonsurgical endodontic retreatment with intentional replantation and extraoral repair to address both the intracanal infection and the inaccessible external defect. Importantly, the observation period was not a continuous 4-year follow-up: the tooth was examined at 2 weeks and then again at 4 years after a substantial undocumented interval. Consequently, transient complications during that interval cannot be excluded. Accurate characterization of ECR is central to treatment planning. The three-dimensional Patel classification incorporates lesion height, circumferential spread, and proximity to the root canal, and CBCT can improve assessment of these features compared with periapical radiography alone [3,4,5]. The absence of preoperative CBCT is a significant limitation of this case because the circumferential extent, true apicocoronal extent, and relationship to the canal could not be quantified preoperatively, preventing formal three-dimensional classification.

The decision to use intentional replantation was based on the subcrestal distal location and the inability to obtain complete visualization and debridement through the intraoral approach. Contemporary ECR literature emphasizes individualized decision making based on lesion accessibility, extent, probing feasibility, symptoms, and periodontal consequences, and current evidence does not establish one universally superior treatment approach [6,7].

Nonsurgical retreatment was performed before intentional replantation to reduce the intracanal microbial load and address the endodontic component of the disease [8,10]. Placement of an intracanal medicament and resolution of pain, swelling, and tenderness before the second appointment indicated control of the acute clinical signs before the surgical procedure was undertaken [13]. The internally accessible part of the ECR defect was debrided at the first visit, whereas the inaccessible distal component was reserved for direct extraoral management. Apical patency could not be achieved in either mesial canal, and the exact cause could not be confirmed. Because apical disinfection and sealing of these canals could not be accomplished predictably by the orthograde route, root end resection, retrograde preparation, and root end filling were incorporated into the extraoral phase [8,10].

Complete management of ECR requires that the resorptive tissue be traced to its external portal of entry and thoroughly removed or inactivated before the defect is restored [1,3,7]. In this case, partial internal debridement was performed first, followed by extraoral access that enabled direct visualization and mechanical debridement of the distal root surface, an approach that would have been difficult to reproduce in situ. This combined internal and external approach was intended to reduce the likelihood of leaving active resorptive tissue in inaccessible areas of the defect. Both the internal and external surfaces of the lesion were subsequently repaired with bioceramic putty to establish a seal between the root canal system, the resorptive cavity, and the periodontal tissues [1,7,10].

Heithersay [1] suggested that mineral trioxide aggregate has favorable properties for repairing resorptive defects, particularly when the restorative material contacts periodontal tissue. Calcium silicate-based materials are similarly favored in contemporary endodontic surgery for their sealing ability, biocompatibility, and potential to support hard-tissue deposition [8]. A fast-setting bioceramic putty may be particularly advantageous during intentional replantation, since conventional MTA can remain vulnerable to displacement or washout while the replanted tooth is mobile during early healing [10]. Nevertheless, clinical evidence specifically evaluating bioceramic putty for the extraoral repair of ECR during intentional replantation remains limited, and most available evidence consists of case reports and small case series [9]. Preserving viable periodontal ligament cells is considered one of the most important biological determinants of healing after intentional replantation [8,10,11]. Atraumatic extraction, minimal root surface handling, prevention of desiccation, and a short extraoral period are therefore emphasized in contemporary protocols [11]. In this case, the root surfaces were continuously irrigated with Pedialyte and the extraoral time was kept below 15 min. Pedialyte has not been established as a preferred periodontal ligament storage medium in the cited intentional replantation literature; its use in this case should be interpreted only as a means of maintaining moisture. Established physiologically compatible media such as saline or Hank’s balanced salt solution have a stronger basis in the literature [8,10,11]. The favorable outcome cannot be used to infer superiority of Pedialyte over these media.

The need for splinting after intentional replantation remains debated, as some authors recommend it only when the replanted tooth demonstrates clinically significant instability [8,10,11,13]. In this case, a flexible stainless steel 0.014 splint was applied from tooth #36 to tooth #38 to maintain stability during the initial period of periodontal healing and was removed after two weeks [8,10]. When splinting is required, a flexible or semirigid technique for a limited period is preferred, as it permits physiologic tooth movement and avoids prolonged rigid immobilization [10,13]. Javed et al. [13] similarly used short-term flexible splinting following intentional replantation of a mandibular first molar and reported complete periapical healing after two years.

Retention, function, periapical healing, periodontal health, and absence of resorptive complications should be considered separately when evaluating intentional replantation [10]. At the 4-year reassessment, the tooth was functional and asymptomatic and the previous periapical radiolucency had resolved. Assessment for ankylosis relied on the clinical mobility/percussion findings, together with the periapical radiograph. Because two-dimensional radiography may not reveal small areas of replacement resorption or ankylosis, the appropriate wording is ‘no clinically or radiographically evident signs’ rather than definitive exclusion. The radiographic definition of healing in this report is qualitative and is based on disappearance of the lesion together with re-establishment of surrounding periradicular structures. Cho et al. [10] reported a cumulative 12-year retention rate of 93%, although the complication-free healing rate was 77% after three years. Mainkar [14] reported an overall survival rate of 89.1% for teeth treated with modern intentional replantation techniques, and Pisano et al. [9] reported an overall survival rate of 86.7%, with no statistically significant difference between the survival of single-rooted and multi-rooted replanted teeth. These findings support intentional replantation as a reasonable tooth-preserving option for selected mandibular molars, despite the greater technical difficulty of atraumatic extraction in multi-rooted teeth [9].

Long-term follow-up is clinically important, since delayed external root resorption, ankylosis, or recurrence of periapical disease may not become evident during short-term review [10]. Although most complications after intentional replantation occur during the first postoperative year, some cases of external root resorption and recurrent periapical radiolucency have been detected after longer observation periods, and follow-up of at least three years has therefore been recommended to identify delayed complications and distinguish short-term retention from stable healing [10]. The absence of clinical or radiographic complications at four years strengthens the interpretation of a favorable outcome in this case, although continued long-term monitoring remains advisable [10]. Published cohorts and systematic reviews of intentional replantation report favorable tooth retention in selected cases, but survival and complication-free healing are not identical outcomes and the evidence is heterogeneous [9,10,11,14]. Evidence specifically concerning ECR is even more limited: a 2023 systematic review found that the available ECR literature consisted of case reports and case series and could not identify a superior treatment approach [7]. Thus, the favorable result in this case supports documentation of intentional replantation as a possible tooth-preserving strategy in selected circumstances but does not establish the efficacy of any individual component of the multimodal treatment.

The patient did not receive the recommended full-coverage crown, despite the extensive previous restoration, endodontic retreatment, cervical defect repair, and extraoral surgical procedures. Although the tooth remained functional at four years, this outcome should not be interpreted as evidence that definitive cuspal coverage is unnecessary after intentional replantation [14]. Mainkar [14] noted that intentionally replanted teeth may require a core, post, and crown depending on the remaining coronal structure and the condition of the previous restoration. The absence of definitive cuspal coverage, the interruption of scheduled follow-up, and the inability to obtain CBCT represent limitations of this case, as they may affect the long-term structural prognosis and reduce the availability of serial clinical and radiographic observations. Root resorption represented only a small proportion of the indications included in the systematic review by Pisano et al. [9], underscoring that evidence for intentional replantation specifically in ECR cases remains limited. Accordingly, this case supports intentional replantation as a potential treatment for inaccessible ECR lesions that cannot be predictably managed in situ, though careful case selection and adherence to biological principles remain essential [8,9].

## 4. Conclusions

At the 4-year reassessment, tooth #37 was functional and asymptomatic and showed favorable periapical healing after combined nonsurgical retreatment, extraoral ECR repair, root end management, and intentional replantation. Because this is a single case treated with a multimodal approach, the contribution of any individual treatment component cannot be determined. In addition, routine follow-up was not continuous between the 2-week visit and the 4-year reassessment, so transient complications during that interval cannot be excluded. This case documents that intentional replantation may be considered as a tooth-preserving option in carefully selected teeth with conventionally inaccessible ECR when other approaches are impractical, provided that periodontal ligament preservation, efficient extraoral treatment, appropriate repair, and long-term monitoring are emphasized [7,9,11].

## Figures and Tables

**Figure 1:**
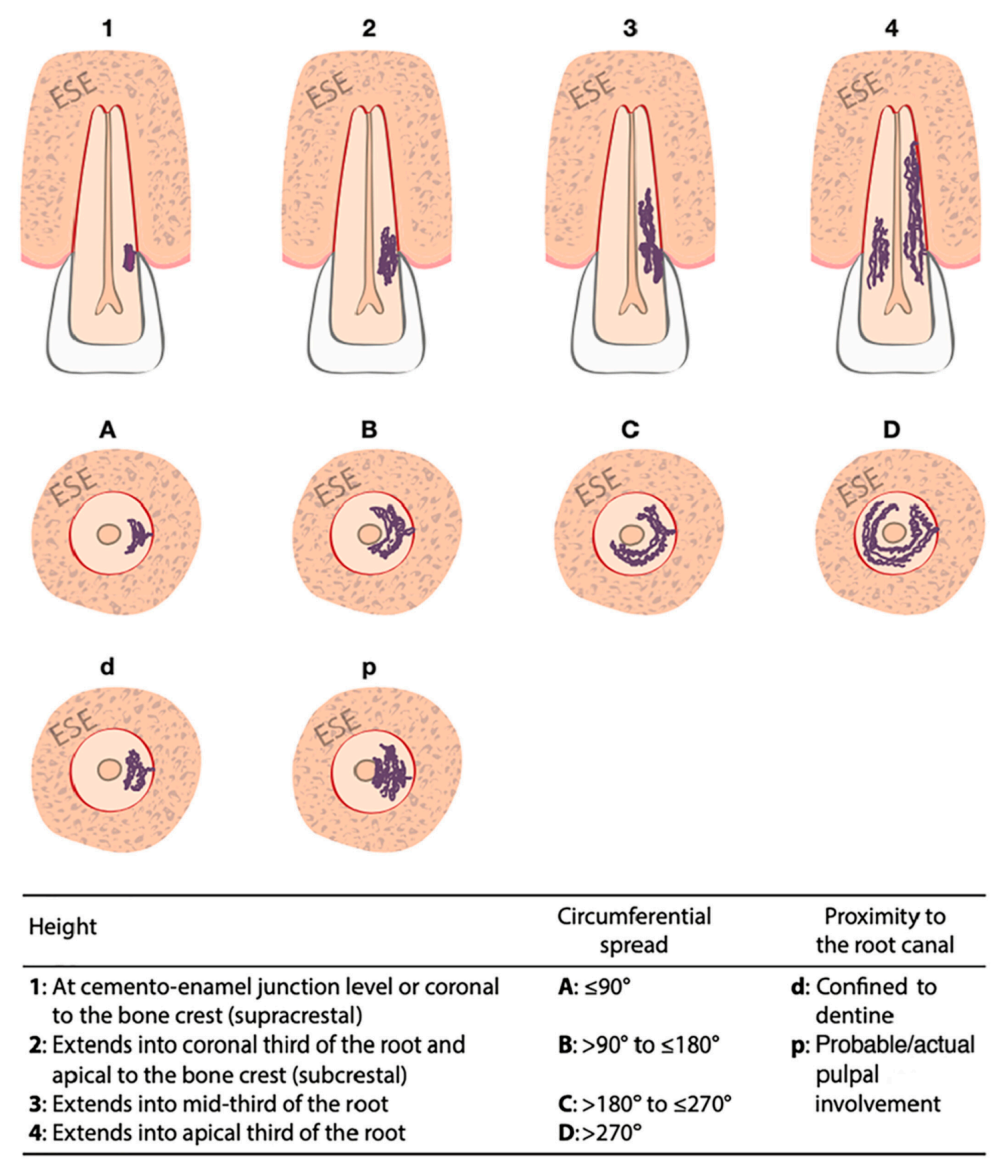
Three-dimensional Patel classification of external cervical resorption according to lesion height (**1**–**4**), circumferential spread (**A**–**D**), and proximity to the root canal (**d**, confined to dentine; **p**, probable pulpal involvement) [4].

**Figure 2:**
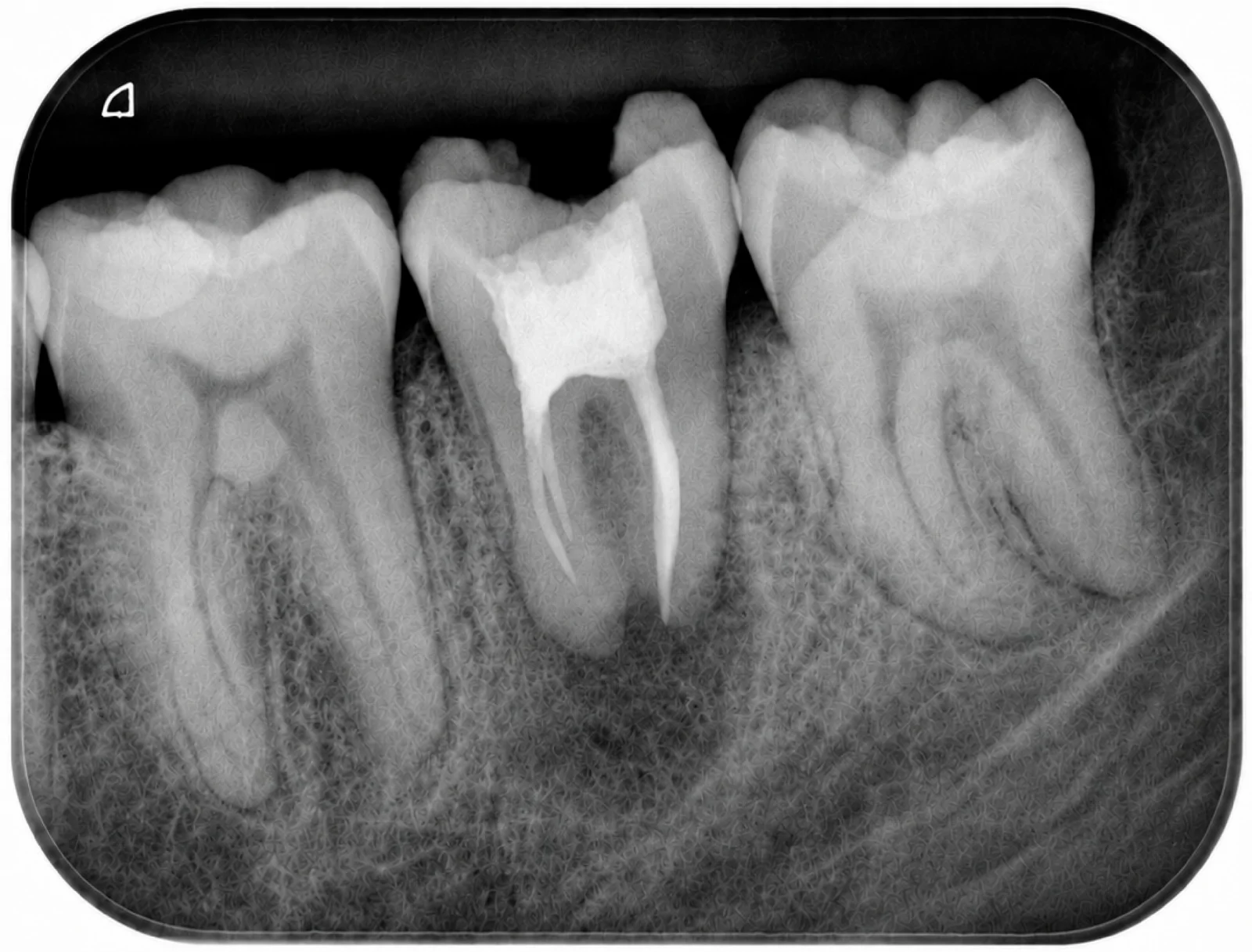
Preoperative periapical radiograph of tooth #37 showing inadequate previous root canal treatment, a defective coronal restoration, a distal cervical resorptive defect extending apical to the crestal bone, and a large periapical radiolucency.

**Figure 3:**
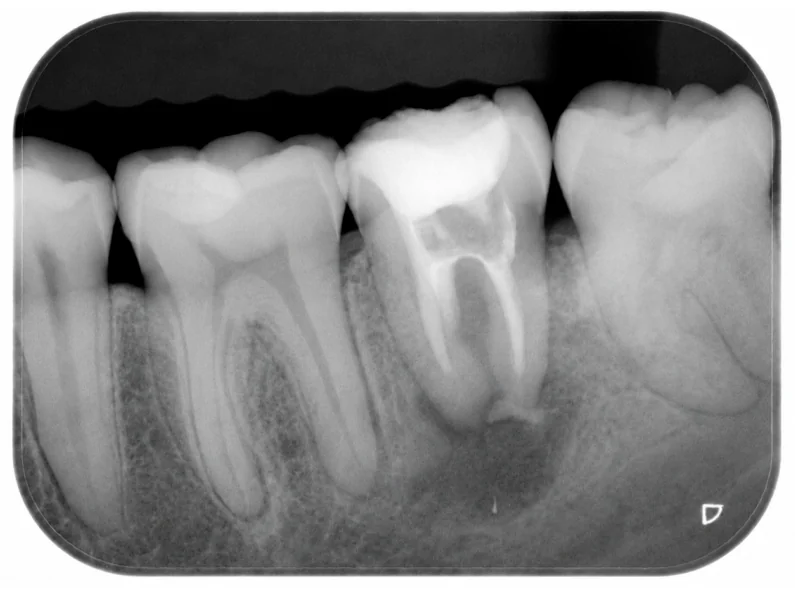
Periapical radiograph obtained after the first treatment visit, showing the calcium hydroxide intracanal dressing and provisional coronal restoration in tooth #37.

**Figure 4:**
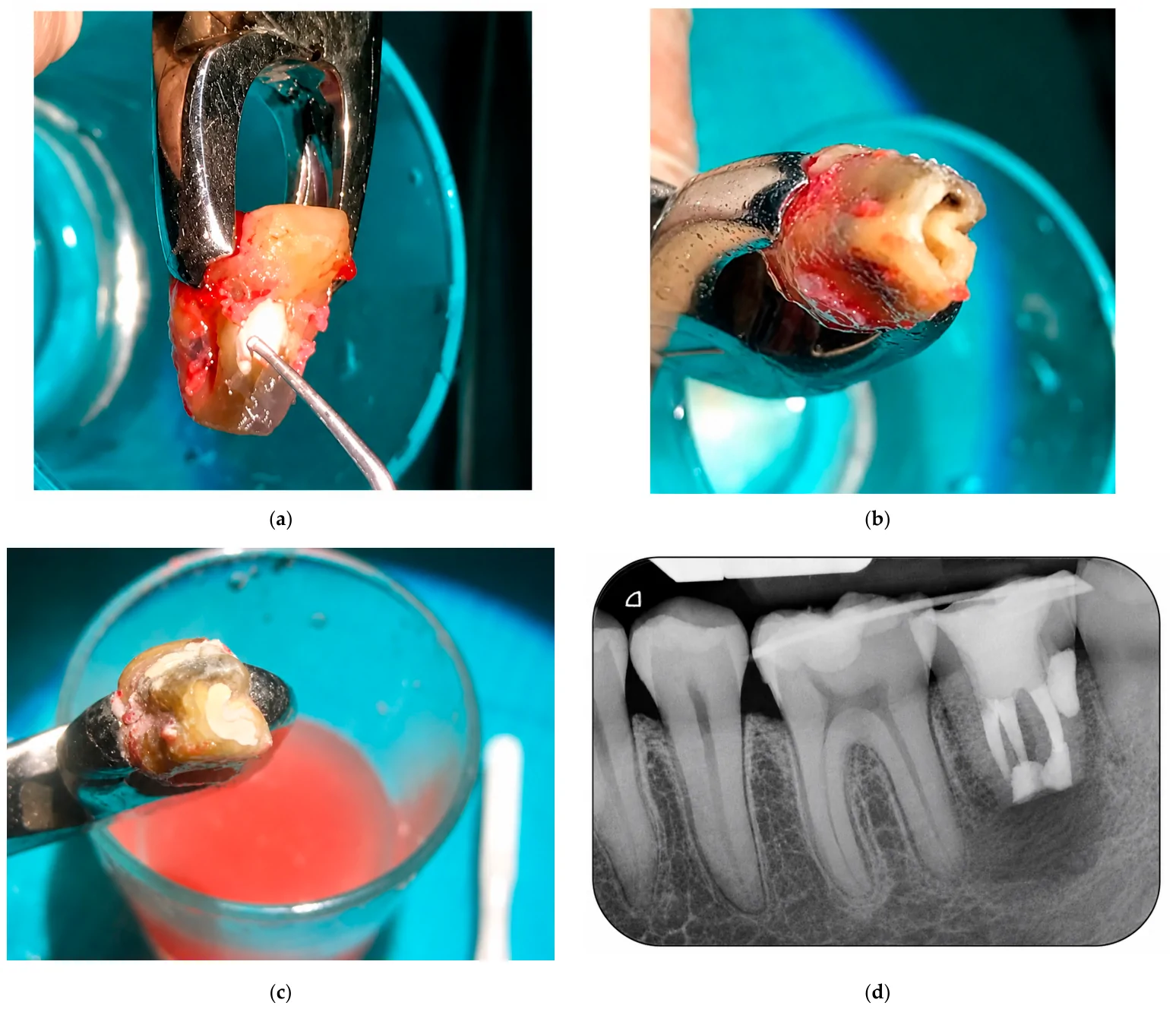
(**a**) Clinical photograph of the extracted mandibular left second molar (tooth #37) during intentional replantation, showing the distal external cervical resorptive defect after debridement and during repair. (**b**) Clinical photograph showing the resected root ends after retrograde preparation. (**c**) Clinical photograph showing the root end cavities after placement of the retrograde repair material. (**d**) Immediate postoperative periapical radiograph after replantation and splinting, showing the tooth repositioned in its socket, completed canal obturation, cervical defect repair, radiopaque root end repair material at the resected mesial and distal apices, and a flexible splint extending from tooth #36 to tooth #38.

**Figure 5:**
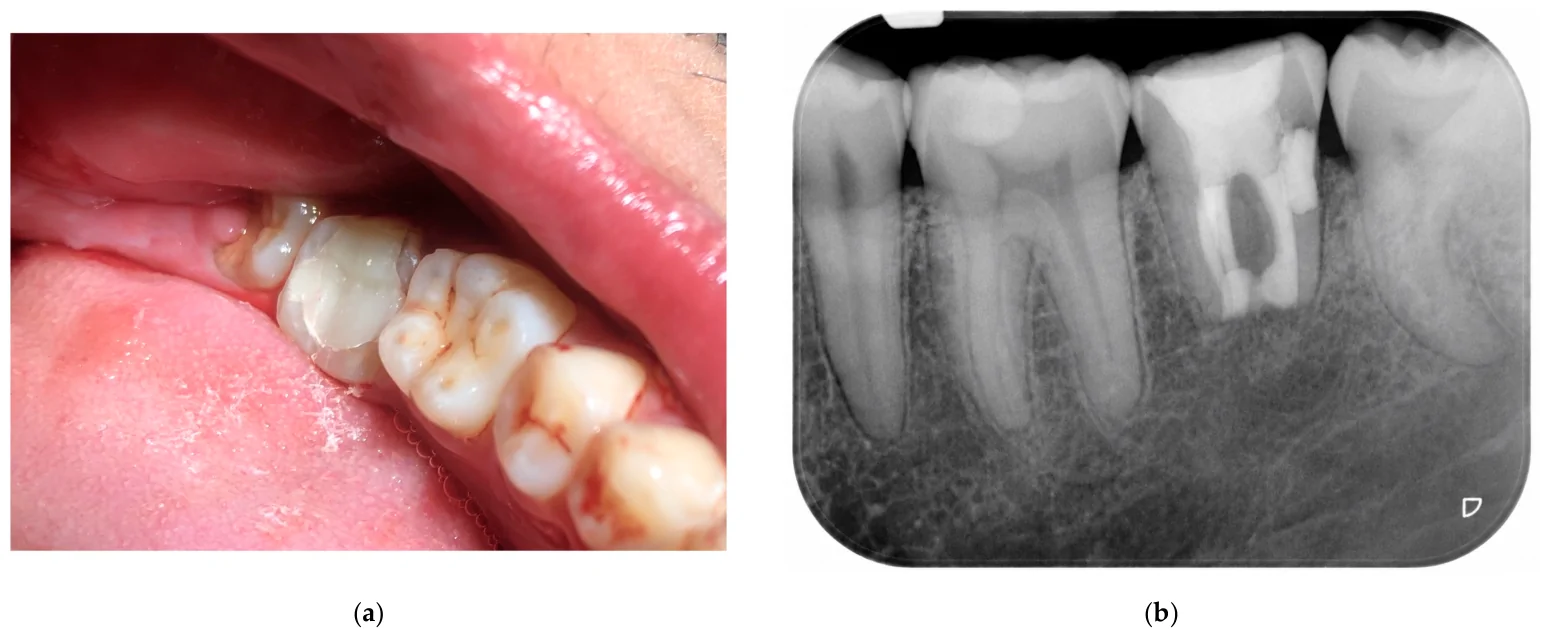
(**a**) Clinical photograph obtained 2 weeks after intentional replantation and removal of the flexible splint, showing tooth #37 maintained in its original position with satisfactory clinical stability. (**b**) Periapical radiograph obtained at the 4-year reassessment, showing resolution of the previous periapical radiolucency and satisfactory periradicular healing, with no clinically or radiographically evident signs of progressive root resorption or ankylosis.

**Table 1:** Clinical timeline.

Time	Clinical Event
Approximately four years before presentation	Root canal treatment performed on the mandibular left second molar.
Presentation	Pain and swelling; fractured restoration; distal cervical resorption; and large periapical radiolucency identified.
First visit	Nonsurgical retreatment initiated; purulent drainage from distal canal; and calcium hydroxide placed.
Second visit	Canals obturated; intentional replantation performed; resorptive defect and root ends repaired extraorally; and flexible splint placed.
Two weeks	Splint removed; tooth clinically stable and asymptomatic.
Four years	Tooth functional and asymptomatic; complete periapical healing; and no evident progressive resorption or ankylosis.

## Data Availability

The data presented in this study are available on request from the corresponding author. The data are not publicly available due to privacy restrictions.

## Abbreviations

The following abbreviations are used in this manuscript:

ECR	External cervical resorption
EDTA	Ethylenediaminetetraacetic acid
